# Primary Diffuse Large B-Cell Lymphoma of the Liver in a Patient with Sjogren Syndrome

**DOI:** 10.1155/2016/2053257

**Published:** 2016-02-22

**Authors:** Vadim Gorodetskiy, Wolfram Klapper, Natalya Probatova, Vladimir Vasilyev

**Affiliations:** ^1^Department of Intensive Methods of Therapy, V.A. Nasonova Research Institute of Rheumatology, Russian Academy of Medical Sciences, Kashirskoye Shosse 34A, Moscow 115522, Russia; ^2^Department of Pathology, Hematopathology Section and Lymph Node Registry, Christian-Albrecht University Kiel and University Hospital Schleswig-Holstein, Arnold-Heller Strasse 3, 24105 Kiel, Germany; ^3^Department of Pathology, N.N. Blokhin Russian Cancer Research Center, Russian Academy of Medical Sciences, Kashirskoye Shosse 24, Moscow 115478, Russia

## Abstract

Sjögren's syndrome (SS) has the highest incidence of malignant lymphoproliferative disorders transformation among autoimmune diseases. We present a case of extranodal high grade lymphoma of the liver in a 52-year-old patient with long history of SS. Lymphoma manifested with sharp significant pain in the right hypochondrium, weakness, and profuse night sweats. Contrast-enhanced computed tomography scan (CT-scan) of the abdomen revealed multiple low density foci with homogeneous structure and clear contours in both lobes of the liver. Histologically, proliferation of medium sized lymphoma cells with round-oval and slightly irregular nuclei with fine chromatin was shown. Immunohistochemical and molecular features of the tumors allowed diagnosis of diffuse large B-cell lymphoma (DLBCL). To exclude secondary liver lesion by non-Hodgkin lymphoma, chest and small pelvis CT-scan, endoscopy of upper and lower gastrointestinal tract and study of bone marrow were performed. After 8 cycles of R-CHOP chemotherapy (rituximab, cyclophosphamide, doxorubicin, vincristine, and prednisone), the complete remission was achieved, which persists after 45 months of follow-up. Primary hepatic lymphomas are extremely rare, and previously only low-grade hepatic lymphomas have been described in SS. To our knowledge, the patient described here represents the first reported case of DLBCL with primary liver involvement in SS.

## 1. Introduction

Sjögren's syndrome (SS) is a chronic autoimmune disease characterized early in its course by lymphocytic infiltration in the salivary and lacrimal glands, resulting in the major manifestations of keratoconjunctivitis sicca and xerostomia [1].

Among autoimmune diseases, SS has the highest incidence of malignant lymphoproliferative transformation, so SS has been considered a crossroad between the autoimmune and lymphoproliferative disorders [2]. SS is associated with a ninefold increase of diffuse large B-cell lymphoma (DLBCL) risk [3]. DLBCL in SS can be nodal or arise in different extranodal sites [4–7].

We describe a case of primary DLBCL of the liver, a rare extranodal lymphoma, in a patient with SS.

## 2. Case Presentation

A 52-year-old woman was admitted to our hospital with severe pain in the right hypochondrium, weakness, and profuse night sweats. On physical examination, the only findings were an increase (4 cm below the costal arch) and sharp tenderness of the right liver lobe and dryness of oral mucosa.

Her past medical history was significant for 32 years of the SS. At the age of 20, she presented with recurrent parotitis. Subsequently, she developed polyarthralgias, Raynaud's syndrome, dry eyes, dry mouth, and difficulty when swallowing without fluid. Contrast X-ray study of the parotid gland showed parenchymal parotitis. A labial minor salivary gland biopsy showed marked focal lymphocytic sialadenitis, with a focus score of 4 (>50 lymphocytes in 4 mm^2^ tissue sample). Schirmer's test was positive (<1 mm in 5 min). Serological testing revealed rheumatoid factor (RF) in 1 : 320 titer (latex agglutination test) and antinuclear antibodies (ANA). Based on clinical, serological, and pathologic features of SS and the absence of radiographic changes of RA, the primary SS was diagnosed. She obtained treatment with glucocorticosteroids, cyclophosphamide, chlorambucil, and topical moisturizing agents.

At admittance, the complete blood count and urinalysis showed no pathological changes. Serum lactate dehydrogenase was increased to 772 IU/L (normal range < 225), alanine aminotransferase to 238 IU/L (normal range < 41), aspartate aminotransferase to 135 IU/L (normal range < 38), alkaline phosphatase to 709 IU/L (normal range < 129), gamma glutamyl transpeptidase to 959 IU/L (normal range < 50), serum fibrinogen to 6 g/L (normal range < 4), and C-reactive protein to 32.2 mg/L (normal range < 5). Blood glucose, bilirubin, creatinine, total protein, albumin, and electrolytes values were within normal limits. Serological testing revealed antinuclear antibody (ANA) in 1 : 320 titer (normal range < 1/160) homogeneous, and with speckled patterns. Rheumatoid factor was absent. Levels of anti-SS-A, anti-SS-B, anticyclic citrullinated peptide antibodies, and antimitochondrial antibodies (AMA) were normal. Levels of serum alphafetoprotein and carcinoembryonic antigen were not elevated. Serology for human immunodeficiency, hepatitis C (HCV), and hepatitis B (HBV) viruses was negative.

**Figure 1:**
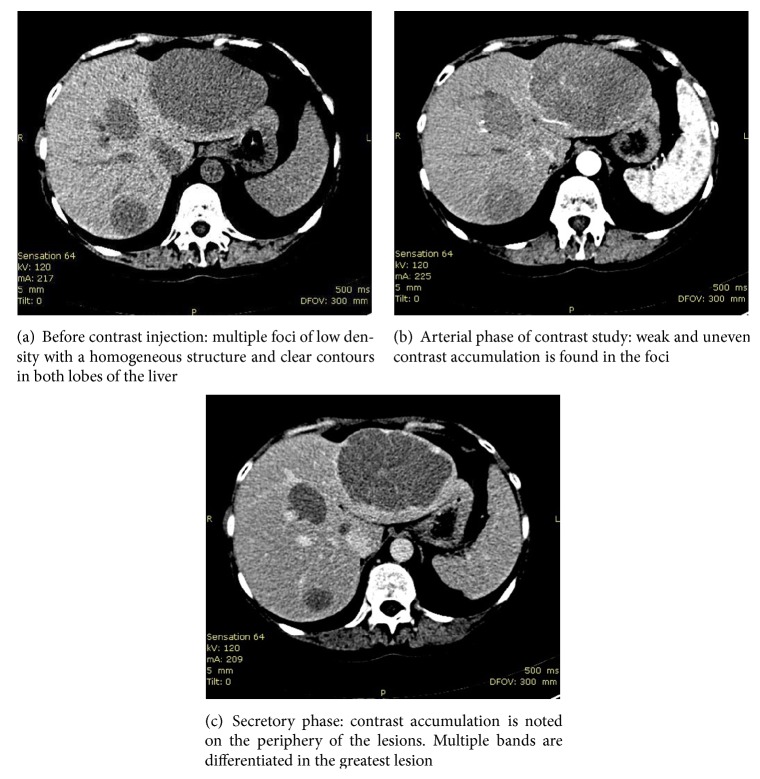
Abdominal CT-scan.

An abdominal contrast-enhanced computed tomography scan (CT-scan) revealed in both lobes of the liver multiple low density foci of homogeneous structure and with clear contours (Figure 1). No other abdominal pathology was found.

**Figure 2:**
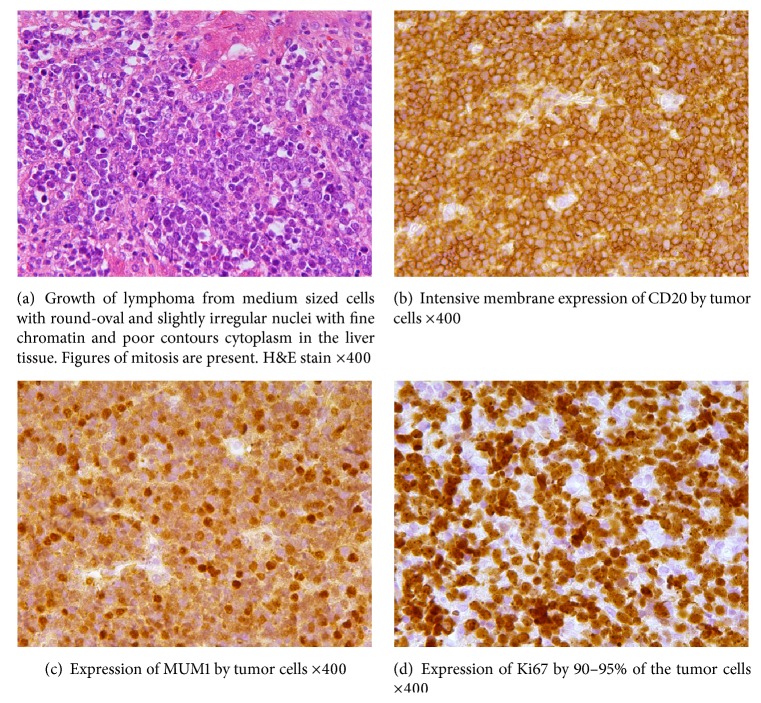
Histological and immunohistochemical study.

Diagnostic laparoscopy was performed with biopsy of tumor node and of visually preserved liver tissue. Liver tissue had no histological signs of inflammation, and tumor node showed proliferation of medium sized lymphoma cells with round-oval and slightly irregular nuclei with fine chromatin and poorly contoured cytoplasm (Figure 2(a)). Immunohistochemically, the tumor cells expressed CD45, CD20 (Figure 2(b)), CD10, MUM1 (Figure 2(c)), PAX5, and bcl-6, and only very weak staining for BCL2, but were negative in reactions with the CD2, CD3, CD4, CD5, CD8, CD30, VS38c, Cyclin–D1, and TdT. Proliferative activity marker Ki-67 was expressed in nearly 95% of tumor cells (Figure 2(d)). EBV RNAs were not detected by hybridization techniques. Chromosomal rearrangements were checked using probes from Vysis/Abbott. No breaks in the BCL2-, BCL6-, MYC- and MALT1-gene were detectable. Additionally, the fusion assay for t(8;14) translocation was negative. In summary, all findings suggested this lymphoma to be DLBCL. To exclude secondary liver lesion by non-Hodgkin lymphoma, chest and small pelvis contrast-enhanced CT-scan, endoscopy of upper and lower gastrointestinal tract and histological and cytological bone marrow studies were performed. No signs of lymphoma in the areas investigated were found. Extranodal DLBCL with primary liver lesion was diagnosed.

After 8 cycles of R-CHOP (rituximab, cyclophosphamide, doxorubicin, vincristine, and prednisone) chemotherapy, the complete remission was achieved, which persists after 45 months of follow-up.

## 3. Discussion

Primary hepatic lymphoma (PHL) was first described in 1965 by Ata and Kamel [8]. In order to define the condition as PHL, liver has to be the only site of lymphoma occurrence or to be involved in a major degree with minimal nonliver disease.

PHL is very rare and constitutes only 0.016% of all non-Hodgkin lymphoma (NHL) cases, and up to date, only about 300 cases of PHL were published in the literature. Of all primary extranodal non-Hodgkin lymphoma cases, only 0.4% occur in the liver [9]. Based on histological and immunohistochemical data, different subtypes of primary lymphoma of the liver were described. The most common NHL variant in the liver is DLBCL, accounting in one study for as large as 71% of all the PHL cases [10].

Etiologic factors associated with PHL are HIV, HBV, HCV, EBV, immunosuppressive therapy, and autoimmune diseases. However, until now, the pathogenesis of PHL is still unclear [11]. Our patient suffered from Sjogren syndrome for a long time and received immunosuppressive therapy, but SS-associated lymphoma risk factors, such as purpura, increased salivary glands, leukopenia, low complement component C4, and cryoglobulinemia, were absent. In addition, hepatitis viruses, HIV, and EBV were negative.

An abdominal contrast-enhanced CT-scan revealed multiple foci in both lobes of the liver that could be considered as hepatocellular carcinoma or metastatic solid tumor lesion of the liver. The high level of LDH and normal serum alphafetoprotein and carcinoembryonic antigen helped in differential diagnosis from hepatocellular carcinoma or metastatic disease. Morphology of tumor lymphocytes and high index of proliferative activity justified differential diagnosis between DLBCL, Burkitt-lymphoma, and lymphoma intermediate between DLBCL and Burkitt-lymphoma. Molecular studies allowed exclusion of Burkitt-lymphoma, and lymphoma intermediate between DLBCL and Burkitt-lymphoma. To stratify DLBCL arising from activated B-cells (ABCs) or from germinal center B-cells (GCBs), we used an algorithm developed by Hans et al. [12]. The tumor cells of DLBCL in our case expressed CD10 and MUM1, and therefore the DLBCL was classified as lymphoma arising from germinal center B-cells.

As far as we know, only 5 cases of primary lymphoma of the liver are described in patients with the Sjogren disease. In three cases, it was extranodal marginal zone B-cell lymphoma of mucosa-associated lymphoid tissue (MALT lymphoma) [13]. In two cases, the subtype of lymphoma was not clearly defined, but these lymphomas progressed slowly and had B-cell immunophenotype [14, 15], allowing for describing them as a low-grade B-cell lymphoma.

Voulgarelis et al. analyzed 54 cases of lymphoma in SS patients and found marginal zone cells lymphoma in 74% and DLBCL in 15% of the patients [16]. The majority of the DLBCL cases in SS patients may be transformed from preexisting marginal zone B-cell lymphomas [17]. In our cohort from 128 patients with Sjogren disease and lymphoma, DLBCL was diagnosed in 19 patients (15%). From them, in 4 histories of MALT lymphoma of parotid gland was found and we therefore could not exclude DLBCL that arose from low-grade B-cell lymphoma. Hypothetically, our patient could have subclinical inflammatory process in the liver, well-recognized complication of SS [18–20], that underwent transformation to DLBCL. However, no clinical, laboratory, or histological evidence of hepatic inflammatory process was noted. No history and morphological signs of preceding MALT lymphoma were found. Immunophenotypically, the cells of DLBCL expressed markers of germinal center B-cells, and no MALT1 gene rearrangements, typical for MALT lymphoma, were detected. This allows us to suppose that DLBCL in our patient arose de novo. It remains unclear how NHL develops in organs that do not typically contain lymphoid tissue. To our knowledge, the patient described here represents the first reported case of DLBCL exclusively involving the liver in SS.
